# Biochar as a UV Stabilizer: Its Impact on the Photostability of Poly(butylene succinate) Biocomposites

**DOI:** 10.3390/polym16213080

**Published:** 2024-10-31

**Authors:** Katerina Papadopoulou, Nina Maria Ainali, Ondřej Mašek, Dimitrios N. Bikiaris

**Affiliations:** 1 Laboratory of Polymer Chemistry and Technology, Department of Chemistry, Aristotle University of Thessaloniki, GR 54124 Thessaloniki, Greece; katerina_1991papa@hotmail.com (K.P.); naina@chem.auth.gr (N.M.A.); 2 UK Biochar Research Centre, School of GeoSciences, University of Edinburgh, Alexander Crum Brown Road, Edinburgh EH9 3FF, UK; ondrej.masek@ed.ac.uk

**Keywords:** UV aging, photoaging, photo-oxidation, poly(butylene succinate), biochar, biocomposites, mechanical properties, UV stability, photostability

## Abstract

In the present study, biocomposite materials were created by incorporating biochar (BC) at rates of 1, 2.5, and 5 wt.% into a poly(butylene succinate) (PBSu) matrix using a two-stage melt polycondensation procedure in order to provide understanding of the aging process. The biocomposites in film form were exposed to UV irradiation for 7, 14, and 21 days. Photostability was examined by several methods, such as Fourier transform infrared spectroscopy (FTIR), which proved that new carbonyl and hydroxyl groups were formed during UV exposure. Moreover, Differential Scanning Calorimetry (DSC) measurements were employed to record the apparent UV effect in their crystalline morphology and thermal transitions. According to the molecular weight measurements of composites, it was apparent that by increasing the biochar content, the molecular weight decreased at a slower rate. Tensile strength tests were performed to evaluate the deterioration of their mechanical properties during UV exposure, while Scanning Electron Microscopy (SEM) images illustrated the notable surface alternations. Cracks were formed at higher UV exposure times, to a lesser extent in PBSu/BC composites than in neat PBSu. Furthermore, the mechanism of the thermal degradation of neat PBSu and its biocomposites prior to and upon UV exposure was studied by Pyrolysis–Gas Chromatography/Mass Spectrometry (Py–GC/MS). From all the obtained results it was proved that biochar can be considered as an efficient UV-protective additive to PBSu, capable of mitigating photodegradation.

## 1. Introduction

In the context of the circular economy, a discernible transition has been observed towards the utilization of more recyclable and sustainable products, coupled with a concerted effort to minimize waste. It is anticipated that plastic waste will reach about 16 million tons by 2060 [1]. The accumulation of plastic waste will take hundreds of years to decompose in the environment. Thus, the impact of conventional fossil-based polymers and disposable products on the environment has led to urgent efforts to replace them with biobased and biodegradable polymers. One of the most interesting biobased and biodegradable aliphatic polyesters that has been intensively studied and commercially produced is poly(butylene succinate) (PBSu) [2,3]. It has excellent characteristics, such as good physical and mechanical properties, chemical resistance, and melt processability, while its properties are comparable to those of worldwide used fossil-derived polymers, such as polypropylene (PP) and low-density polyethylene (LDPE) [4]. Furthermore, PBSu is a biobased polymer due to the biomass-based source of its monomers (succinic acid and butanediol) [5] and its biodegradability under certain conditions. Regarding its advantages, PBSu has a lot of promise as a new possible sustainable material in agriculture applications, such as in mulch films [6,7,8,9].

The valorization of biomass is a process promoting new ways to create sustainable alternatives. Considering circular economy principles, pyrolysis is a process to convert biomass into three different sources, including synthetic gas, oil, and biochar. Biochar (BC) is a byproduct of biomass pyrolysis, especially of the production of biofuels. It is an environmentally friendly carbon source with remarkable characteristics such as a stable structure, high specific surface area, high thermal stability, and high carbon content [10,11,12]. The properties of biochar vary based on the feedstock of the biomass and the pyrolysis conditions [13]. Furthermore, BC changes the color of the composites to black. The black color, as well as its carbonaceous and stable structure, has the ability to absorb UV light [14]. These properties are comparable to the relatives of fossil-based fillers (e.g., carbon black, graphene, etc.) and, thus, BC is capable to replace them [15]. Among other environmental applications of biochar, such as in wastewater treatment and soil remediation [10,16,17], the incorporation of biochar as a filler in biobased polymers has only been explored in the last few years [18,19,20]. In our previous studies [21,22,23], we investigated the effect of biochar’s incorporation into PBSu polymeric matrix on the properties of the final materials, such as mechanical properties, thermal properties, and electrical conductivity. For this reason, it is important for a constructive life cycle assessment of biobased and biodegradable polymers to study further the effects of photoaging phenomena.

The photodegradation behavior of polymers is a crucial process due to its close association with the longevity of the polymers and the generation of microplastics (MPs). Microplastics are plastic fragments with dimensions <5 mm, which are formed mainly after the degradation of larger plastic items under the action of several physiochemical mechanisms occurring in the environment, such as UV irradiation, heating, moisture, and micro-organisms attack. Photoaging is the most significant agent of polymer breakdown due to its detrimental effects on material properties. When polymer chains or additives absorb UV light, radicals are formed; these radicals interact with oxygen and induce degradation of the polymer chains. In recent years, the aging effects of polyolefins such as polyethylene (PE) and polypropylene (PP) have been investigated [14,15,16]. Given the importance of understanding polymer aging, the photodegradation stability of PBSu polymers is crucial for outdoor agricultural applications, such as mulch films. Therefore, studying the photodegradation of PBSu and its composites is vital to predict aging effects—including exposure to daylight, solar ultraviolet (UV) radiation, thermo-oxidative degradation, and biodegradation—that provoke crack propagation and fragmentation. The photo-oxidation degradation mechanism of PBSu has been investigated by Carroccio et al. [17]. It is understood that PBSu absorbs UV light during exposure, leading to cleavage of ester linkages and C–C bonds in the polymer matrix, as evidenced by changes in the surface properties of the polymer. Additionally, oxidation of PBSu results in depolymerization, forming intermediate products such as alcohols, ketones, peroxides, and hydroperoxides [18].

Stabilizers can slow down the material’s degradation caused by UV radiation by protecting it from UV damage and preventing the chemical reactions that occur during photodegradation. There are many UV stabilizers such as phenolic antioxidants, light stabilizers, inorganic UV screeners (carbon nanotubes, graphene, TiO_2_), phenolic UV absorbers, etc. [24,25,26]. Apart from being a filler, biochar can also be used as a UV stabilizer. The black color of biochar, among other things, could decrease the material sensitivity to UV exposure. Botta et al. studied the photooxidative resistance on poly(butylene adipate–co–terephalate) (PBAT) with BC content by spectroscopic analysis. The results show a delay in the photodegradation process of the composites [27]. Furthermore, in the study of Wang et al., composites of BC with high-density polyethylene (HDPE) and maleated polyethylene (MAPE) were prepared by extrusion. The use of BC improved the photostability of the composites [28]. To the best of our knowledge, the photostability of PBSu/BC composites has not yet been investigated by the researchers. In this work, PBSu and its biocomposites containing 1, 2.5, and 5 wt.% biochar were exposed to artificial UV radiation for 7, 14, and 21 days and their properties were investigated by GPC, FT-IR, DSC, Py–GC/MS, and SEM, whereas the mechanical performance of the materials was also evaluated in order to provide a complete understanding of the photodegradation mechanism.

## 2. Materials and Methods

### 2.1. Materials

Succinic acid (SA) (purum 99+%) and titanium isopropoxide (≥97%) (Tis) catalysts of analytical grade were obtained from Sigma–Aldrich Chemical Co (Saint Louis, MO, USA). 1,4-Butanediol (BD) (Purity: >99%) was purchased from Alfa Aesar (Haverhill, MA, USA). The BC used in this work was produced by intermediate pyrolysis of pelleted miscanthus straw at 700 °C (MSP 700) in a pilot-scale rotary kiln at the UK Biochar Research Centre (UKBRC) [29,30]. BC was received grinded in large (10–15 µm) and small (100–200 nm) particle sizes. This was confirmed from SEM micrographs in our previous work [21]. It was dried overnight in the oven at 80 °C under a vacuum before every single use. All other reagents were of analytical grade.

### 2.2. Synthesis of PBSu Biocomposites

PBSu was synthesized by the two-stage melt polycondensation method (esterification and polycondensation) using SA and BD in a molar ratio of 1/1.1, as was described in our previous work [21]. In a similar way, PBSu/BC biocomposites have been produced by in situ polymerization containing 1, 2.5, and 5 wt.% BC. To produce a uniform and fine filler dispersion, BC was first added in BD and dispersed with ultrasonication for 5 min. Thereafter, the dispersion was introduced with SA in the glass batch reactor for the polymerization procedure to take place.

### 2.3. Preparation of Films

Biocomposites in the form of thin films with thickness 350 ± 30 μm (7 films for each sample) were prepared using an Otto Weber, Type PW 30 hydraulic press connected with an Omron E5AX Temperature Controller. The temperature was set at 160 °C in order to melt the whole mass of the sample and then the materials were slowly cooled. From these films, dumb-bell-shaped tensile test specimens (central portions 5 × 0.5 mm thick, 22 mm gauge length) were cut in a Wallace cutting press. Prior to the measurements, the samples were conditioned at 25 °C in a 50 ± 5% relative humidity environment for 48 h.

### 2.4. UV Irradiation

The prepared films were exposed at a distance of 25 cm from the lamp. The irradiation intensity was about 500 W/m^2^ at an area of 35 × 45 cm^2^. The specimens were irradiated continuously for 7 h, remaining at room temperature for 7 h, and irradiated again. In order to irradiate both surfaces, the films were flipped once every day. After 7, 14, and 21 days of UV exposure, the films were removed from the chamber and the photodegradation effect was evaluated by using GPC, FTIR, mechanical properties, DSC measurements, and SEM. Furthermore, the mechanism of photodegradation was also investigated using Py–GC/MS.

### 2.5. Gel Permeation Chromatography–Size Exclusion Chromatography (GPC/SEC)

The molecular weight of the materials was determined using gel permeation chromatography–size exclusion chromatography (GPC/SEC) analysis. An Agilent 1260 Infinity II LC system (Agilent Technologies, Santa Clara, CA, USA) equipped with an isocratic G7110B pump, an automatic vial sampler G7129A, a Refractive Index Detector (RID), G7162A, and a PLgel 5 µm (50 × 7.5 mm) guard column combined with 2 PLgel 5 µm (300 × 7.5 mm). MIXED-C columns were employed for the investigation of the molecular weights. For the calibration curve, poly(methyl methacrylate) (PMMA) standards were facilitated. The prepared solutions had a concentration of 3 mg/mL and were filtered through 0.45 µm pore size PTFE filters. The injection volume was 20 µL and the total elution time of each sample was 30 min. The temperature of the columns and the RID were both set at 40 °C.

### 2.6. Fourier Transform Infrared Spectroscopy (FTIR)

FTIR spectra prior to and during UV exposure (7, 14, 21 days) were obtained using a Perkin-Elmer FTIR spectrometer, model Spectrum One (Dresden, Germany). The IR spectra of these films were obtained in absorbance mode and in the spectral region of 450–4000 cm^−1^ using a resolution of 32 co-added scans. Samples were in the form of thin films with thickness of approximately 150 μm (one film for each material) and placed in aluminum holders with the appropriate dimensions of the spectrometer’s accessory.

### 2.7. Differential Scanning Calorimetry (DSC)

Thermal analysis of the composites prior to and upon UV radiation was performed using a Differential Scanning Calorimeter (Perkin Elmer, Pyris Diamond, Waltham, MA, USA), calibrated with Indium and Zinc standards. For each measurement, a sample of 6.0 ± 0.2 mg was used, placed in a sealed aluminum pan, and heated to 150 °C at a scanning rate of 20 °C/min in an inert atmosphere (N_2_ 50 mL/min) and held at this temperature for 2 min in order to obtain the thermal characteristics caused by specific heat treatment, such as aging. Afterwards, the sample was cooled down to 20 °C at a cooling rate of 10 °C/min, held at 20 °C, and heated again to the abovementioned temperatures with a heating rate of 20 °C/min.

### 2.8. Pyrolysis–Gas Chromatography/Mass Spectrometry Analysis (Py–GC/MS)

For Py–GC/MS analysis of the PBSu/BC composites before and throughout the UV aging testing, a very small amount of each material was “dropped” initially into the “Double-Shot” EGA/PY 3030D Pyrolyzer (Frontier Laboratories Ltd., Fukushima, Japan) using a CGS-1050Ex carrier gas selector. For pyrolysis analysis (flash pyrolysis), each sample was placed into the sample cup, which afterward was placed into the Pyrolyzer furnace. The pre-selected pyrolysis temperature set at 450 °C, while the GC oven temperature was programmed at 50 °C for 2 min, followed by a stepped increase to 200 °C with a heating rate of 5 °C/min, where it was held for 8 min, and then the temperature was increased at 300 °C by a rate 20 °C/min, where it was held for 5 min. Sample vapors generated in the furnace were split (at a ratio of 1/50), a portion moved to the column at a flow rate of 1 mL/min at pressure 53.6 kPa, and the remaining portion exited the system via the vent. The pyrolyzates were separated using the temperature-programmed capillary column of a Shimadzu QP-2010 Ultra Plus (Kyoto, Japan) gas chromatogram and analyzed by the mass spectrometer MS-QP2010SE of Shimadzu (Kyoto, Japan). An Ultra-ALLOY^®^ metal capillary column from Frontier Laboratories LTD (Fukushima, Japan) was used containing 5% diphenyl and 95% dimethylpolysiloxane stationary phase, with column length 30 m and column ID 0.25 mm. For the mass spectrometer, the following conditions were used: ion source heater 200 °C, interface temperature 300 °C, vacuum 10^−4^–10^0^ Pa, m/z range 40–500 amu (atomic mass unit), and scan speed 10,000. The ion gas chromatograms and spectra retrieved by each experiment were subjected to further interpretation through Shimadzu and Frontier post-run software. The chromatogram and spectra retrieved by each experiment were subject to further interpretation through Shimadzu (NIST11.0) and Frontier (F-Search software 4.3) post-run software. Identification was recognized depending on the similarity percentage (minimum value of 80%) between average mass spectra on the entire chromatogram.

### 2.9. Mechanical Properties

Measurements of the mechanical properties of the prepared specimens were performed Instron 3344 Dynamometer (Norwood, MA, USA) in accordance with ASTM D638 method, using a crosshead speed 5 mm/min. The values of tensile strength at break, elongation at break, and Young’s Modulus were determined. At least five specimens were tested for each sample and the average values are reported.

### 2.10. Scanning Electron Microscopy (SEM)

SEM was carried out using a JEOL JMS 760F (Jeol, Freising, Germany) scanning microscope operating at 10–20 kV equipped with an energy-dispersive X-ray Oxford ISIS 300 microanalytical system. For this study, the surfaces of the films prior to and during UV radiation were studied. Regarding sample preparation, all surfaces were coated with carbon black in order to avoid charging under the electron beam.

## 3. Results and Discussion

### 3.1. Molecular Weight of PBSu and Its Biocomposites

An analysis of the molecular weight evolution of neat PBSu and its biocomposites before and during UV exposure was carried out by GPC. Table 1 shows the changes in molecular weight at different irradiating times.

**Figure 1:**
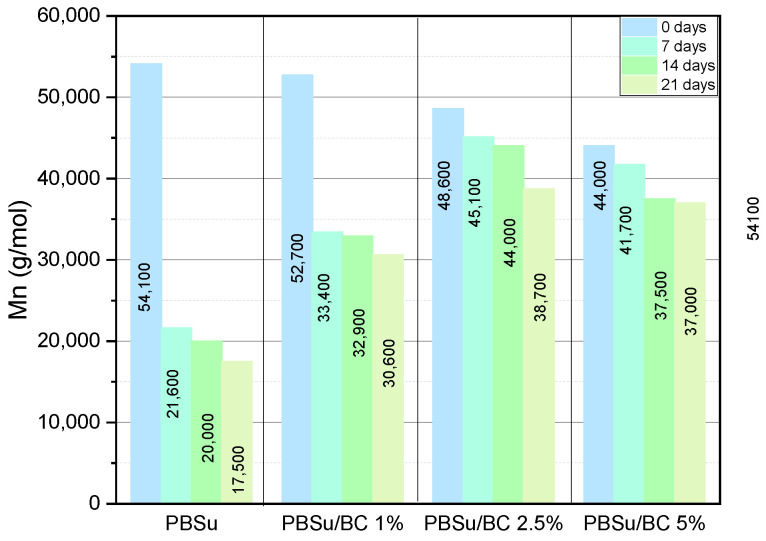
Molecular weight of PBSu and its composites during UV exposure.

At first glance, all samples display a reduction in molecular weight during the aging test, which was expected since decomposition and radical chain scission takes place. However, incorporating biochar into the PBSu matrix protected the samples from the photodegradation process. As can be seen from Table 1, for neat PBSu, the reduction in Mn is 67.6% after 21 days of the aging test. With adding 1 wt.% biochar, the M_n_ decline is about 41.9%. It is apparent that in increasing the biochar content to 2.5 and 5 wt.%, Mn decreases with lower rate, and the reduction is about 20.3% and 15.9%, respectively. Figure 1 displays the comparison prior to and upon UV irradiation. This is a first proof that the addition of biochar protects PBSu from decomposition during UV irradiation. In the study of Zhang et al., the same results were confirmed by incorporating 2-hydro-4-(2,3-epoxypropoxy) benzophenone (HEPBP) moieties in PBSu polymer chains. The decline of the molecular weight by adding HEPBP content into PBSu matrix is lower than that of neat PBSu. Thus, the incorporation of HEPBP delayed the photodegradation process [31].

### 3.2. Chemical Structure Changes

**Figure 2:**
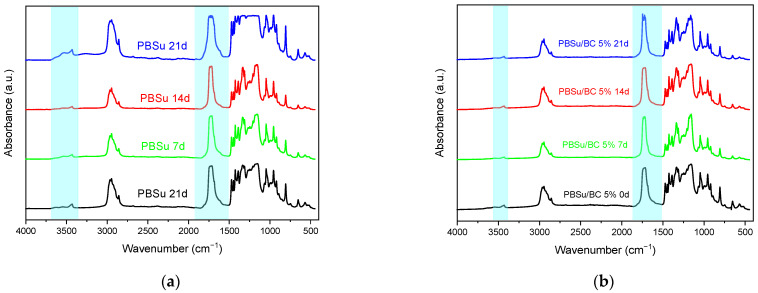
Stacked FT-IR spectra of (**a**) neat PBSu and (**b**) PBSu/BC 5 wt.% upon different UV radiation times.

FT-IR spectroscopy has confirmed the changes in chemical structure upon photodegradation. The irradiated surfaces of the films have noticeable changes on their spectra, when measured by FT-IR spectroscopy. Figure 2 displays the spectra of neat PBSu and its biocomposites with 5 wt.% biochar at different (0, 7, 14, 21 days) exposure times.

**Figure 3:**
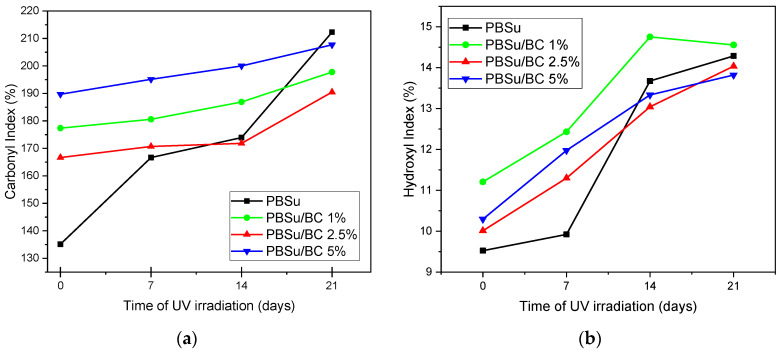
(**a**) Carbonyl index and (**b**) hydroxyl index of PBSu and its composites during UV exposure for 7, 14, and 21 days.

The bands at 1716 cm^−1^ are attributed to the stretching vibration of the ester group (carbonyl groups), while the bands in the range of 3400–3700 cm^−1^ correspond to the stretching of hydroxyl groups of the polyester (hydroxyl/hydroxyperoxide groups). The broad bands in the range of 3000–2800 cm^−1^ are related to the stretching and bending vibration of the C–H bonds. These bands are requisite as the reference peaks in the identification of the photodegradation products [32,33]. It is worth noting that a new band, which indicates the formation of hydroperoxides during UV irradiation, is centered at 3431 cm^−1^ [34]. In comparison to the spectra of neat PBSu, the spectra of the PBSu/BC 5 wt.% biocomposite display less changes at the characteristic bands while the new band at 3431 cm^−1^ does not appear. Carbonyl index (C.I.) and hydroxyl index (H.I.) are calculated and summarized in Figure 3. The area ratio of carbonyl absorbance at 1716 cm^−1^ to the reference at 2946 cm^−1^ is defined as the carbonyl index (Equation (1)); the area ratio of hydroxyl absorbance at 3431 cm^−1^ to the reference 2946 cm ^−1^ is defined as the hydroxyl index (Equation (2)). Generally, calculating C.I. and H.I. during photodegradation is employed to measure not only the oxidation and the decomposition of the polymers, but also to see the effectiveness of stabilizing additives [35].

(1) $$C.I.=\frac{A_{C=O}}{A_{C-H}\;}\times 100$$

where *C.I.*: carbonyl index; *A_C_*_=*O*_: area of peaks attributed to carbonyl groups at 1716 cm^−1^; *A_C-H_*: area of reference peak at 2946 cm^−1^.

(2) $$H.I.=\frac{A_{-OH}}{A_{C-H}}\times 100$$

where *H.I.*: hydroxyl index; *A_-OH_*: area of peaks attributed to hydroxyl group at 3431 cm^−1^; *A_C-H_*: area of reference peak at 2946 cm^−1^.

The acquired indices are shown in different graphs (Figure 3) for pure PBSu and its biocomposites. Figure 3a shows the increase in the values of C.I. Especially for the neat PBSu graph, a steep tendency in the increase in the values of C.I. can be observed. The increase in C.I. indicates the formation of ketones and aldehydes as early steps of degradation and further the formation of hydroperoxides and carboxylic acids [34,36]. It seems that the profile of the increase in C.I. is softer for the PBSu composites. Moreover, the same behavior is also shown for the H.I. values (Figure 3b), where the values of PBSu neat and PBSu/BC 1 wt.% are increased steeper than in the cases of the PBSu/BC 2.5 wt.% and 5 wt.% composites. According to the literature, biochar contains a large amount of carbon and it has a black color, which are crucial factors for absorbing UV light [37]. Similar results have also been reported for carbon nanotubes and graphenes [25,27,38,39].

### 3.3. Differential Scanning Calorimetry (DSC)

From the DSC analysis, information about the thermal properties of the studied samples were obtained. All results prior to and upon artificial UV aging of the samples, including melting temperature (T_m_), crystallization temperature (T_c_), and degree of crystallinity (X_c_), are listed in Table 2. From Table 2, it seems that the melting temperatures as well as crystallization temperatures of neat PBSu decrease progressively during photo-irradiation process from 116 °C to 114 °C and from 71 °C to 47 °C, respectively.

**Figure 4:**
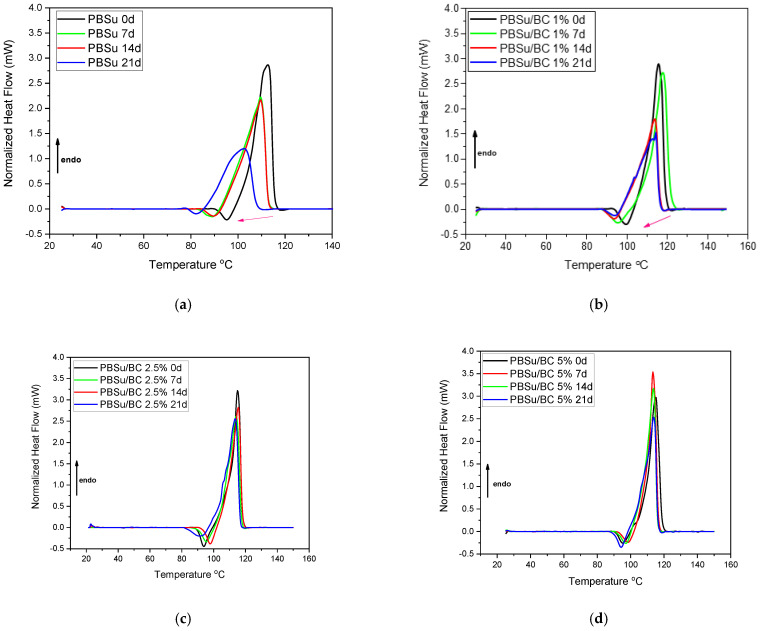
Comparative DSC heating traces, mainly showing the endothermic peaks related to melting of (**a**) PBSu neat, (**b**) PBSu/BC 1 wt.%, (**c**) PBSu/BC 2.5 wt.%, and (**d**) PBSu/BC 5 wt.%, during different days of UV exposure.

From the thermograms of neat PBSu (Figure 4a), it is apparent that there is a progressing shift in the melting point to lower temperatures. Furthermore, it is worth noting that the peaks become constantly wider during UV exposure time. This happens due to the lower molecular weight, resulting in the creation of smaller crystals with fewer imperfections.

In the case of PBSu/BC biocomposites, it is apparent that the reduction in crystallization temperature decreases with increasing the biochar content into the polymer matrix, and finally, the crystallization temperatures remain mostly stable during UV exposure. Given that the Mn values are in no case extremely high, the significant drop of crystallization temperature of pristine PBSu can be expected during aging due to the shortening of the polymer chain and, thus, chain scission, decreasing the average molecular weight. Furthermore, the heat of fusion for pristine PBSu increases as the irradiation time proceeds. The increase in crystallinity is an indirect effect of the polymer chain scission in the amorphous regions allowing the free segments to crystallize within the polymer matrix. An increase in crystallinity has also been observed for the PBSu/BC 1 wt.% over the first 7 days and then, for longer exposure time periods, it decreases and becomes stable. This slight increase in crystallinity can be explained as follows. It could be proposed that the incorporation of the biochar fillers at the nanosized level possibly act as a nucleating agent at 1 wt.% loading, despite the moderate simultaneous lowering of Mn due to the in situ polymerization. After the increase in crystallinity of PBSu and PBSu/BC 1 wt.% irradiated samples, there is a reduction in their crystalline fraction, most probably originating from the further suppression of Mn. The composites with 2.5 wt.% and 5 wt.% biochar present stable crystallinity during the exposure time in UV light. This can be attributed to the presence of heterogeneous nuclei (biochar) in PBSu matrix. As can be noticed, biochar at higher than 1 wt.% loading inhibits chain scission reactions, a fact which may be attributed to the absorbing of UV radiation.

### 3.4. Pyrolysis–Gas Chromatography/Mass Spectrometry Analysis (Py–GC/MS)

Analytical pyrolysis has been extensively explored in the field of polymer decomposition as it provides structural information about macromolecules through the analysis of their evolved thermal degradation products [40]. Each group of molecules produces distinct compounds upon pyrolysis, which serve as indicators of the molecular contributions to the sample and provide valuable structural information on the sample’s chemical composition [41,42]. As for Py–GC/MS analysis, it involves the thermal decomposition of polymer samples in an inert atmosphere (e.g., He), generating volatile products that are subsequently separated and identified using gas chromatography and mass spectrometry. This technique provides a comprehensive analysis of the thermal degradation products, thereby offering insights into the molecular changes occurring within the polymer matrix. In this context, Py–GC/MS was employed not only to elucidate the primary depolymerization mechanisms for the studied polymers and the impact of photo-irradiation on their degradation pathways, but also as an indicative tool for evaluating their oxidation time. One of the primary objectives of this study was to determine whether the incorporation of varying biochar contents affects the thermal degradation profile of PBSu composites during UV aging. By utilizing Py–GC/MS, we could identify specific degradation products and monitor any shifts in their profiles, thus elucidating the role of biochar in enhancing UV stability. Through the analysis of total ion chromatograms (TICs) and corresponding mass spectra, we can detect alterations in the depolymerization mechanisms attributable to different amounts of biochar. Furthermore, by comparing the evolution of degradation products over time, we can evaluate the efficacy of biochar as a UV stabilizer.

As already reported in the literature, the decomposition mechanism of PBSu primarily involves β-hydrogen scission or β-decomposition of the polymer backbone, characterized by a six-membered cyclic transition state [23,43,44,45] and to a less extent α-hydrogen scission paths. Hydroxyl-terminated compounds can form either through acyl–oxygen (C–O) homolytic scission or hydrolysis of the β-decomposition products [46]. A limited number of degradation products attributed to the homolytic α-hydrogen scission pathway could also be detected in some cases.

**Figure 5:**
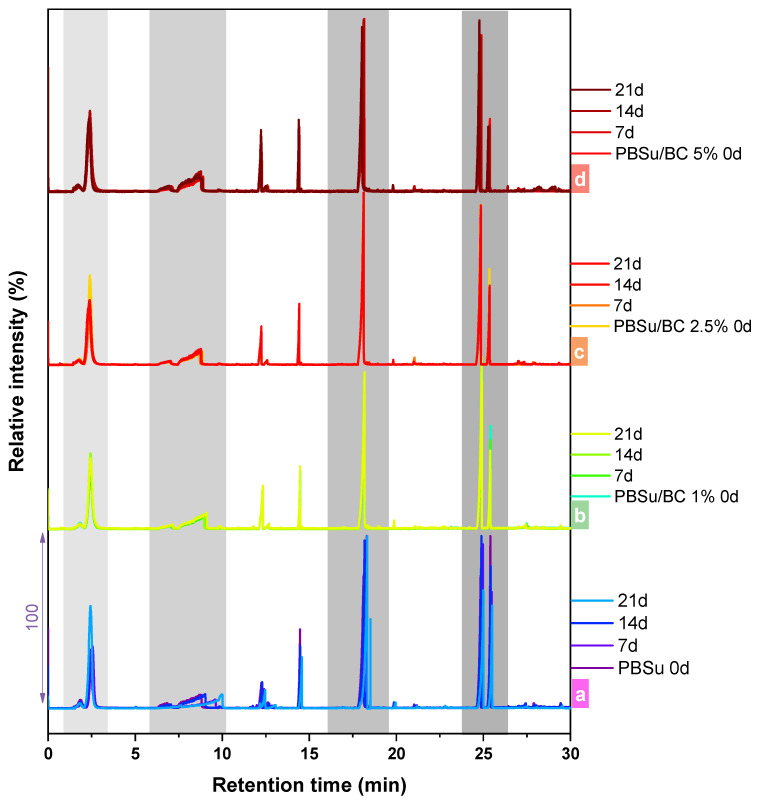
Total ion chromatograms (TICs) of (**a**) neat PBSu with different contents in biochar, (**b**) 1 wt.%, (**c**) 2.5 wt.%, and (**d**) 5 wt.%, during UV exposure testing for different intervals (7, 14, 21 days).

Figure 5 depicts the total ion chromatograms (TICs) of neat PBSu incorporating different contents of biochar, before and throughout UV exposure for certain intervals after flash pyrolysis at 450 °C. Table 3 and Tables S1–S3 (please check also Supplementary Materials) report the characteristic pyrolysis products formed during thermal degradation of the PBSu, PBSu/BC 1 wt.%, PBSu/BC 2.5 wt.%, and PBSu/BC 5 wt.%, respectively, with their interpretation based on the mass spectra. As previously reported by our team concerning the thermal degradation pathway of PBSu/BC samples [23], the TICs of the studied composites showed only minor differences with the neat PBSu due to the incorporation of the filler, as the chromatograms of virgin composites were nearly the same upon initial inspection. The evolved products (Table 3 and Tables S1–S3) were detected in a similar pattern and were identical. In our study of PBSu/biochar biocomposites with different biochar ratios (1, 2.5, and 5 wt.%) subjected to UV exposure for 7, 14, and 21 days, we observed some shifts in retention times and changes in peak intensities in the recorded TICs for both neat PBSu and PBSu/BC 5 wt.% sample.

**Figure 6:**
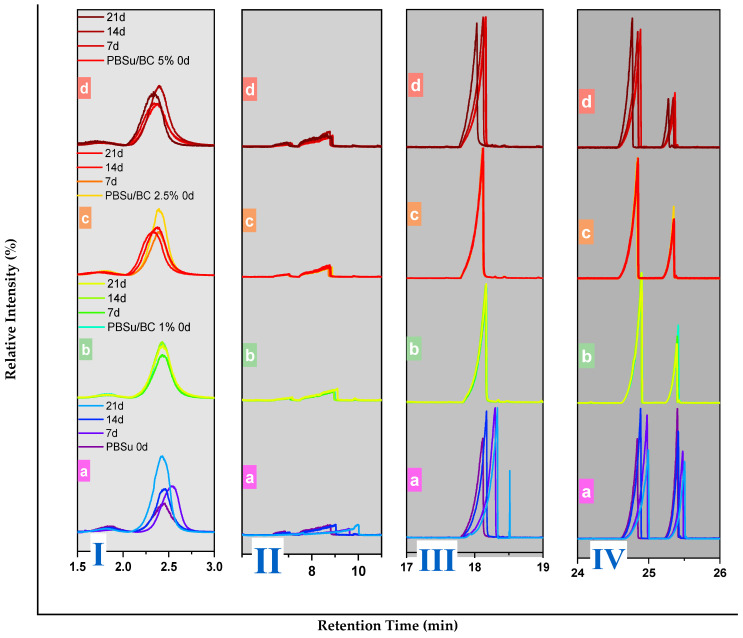
Enlarged TICs for (**I**) Rt = 1.5–3 min, (**II**) Rt = 6–11 min, (**III**) Rt = 17–19 min, and (**IV**) Rt = 24–26 min. As in Figure 5, the diagram’s annotations are the following: (a) PBSu, (b) PBSu/BC 1 wt.%, (c) PBSu/BC 2.5 wt.%, and (d) PBSu/BC 5 wt.% for untreated samples and during UV testing.

Aiming to better demonstrate the changes occurring at specific retention time slots, TICs of the studied samples with zoomed regions are presented in Figure 6. These shifts could be attributed to UV-induced chemical modifications such as oxidation, chain scission, and cross-linking reactions, which alter the chemical structure of the polymer and consequently could possibly change the formation of degradation products. Notably, the TICs for PBSu/BC biocomposites with 1% and 2.5 wt.% biochar remained almost stable during UV aging, indicating that lower concentrations of biochar are effective in maintaining the integrity of the polymer matrix under UV exposure.

**Figure 7:**
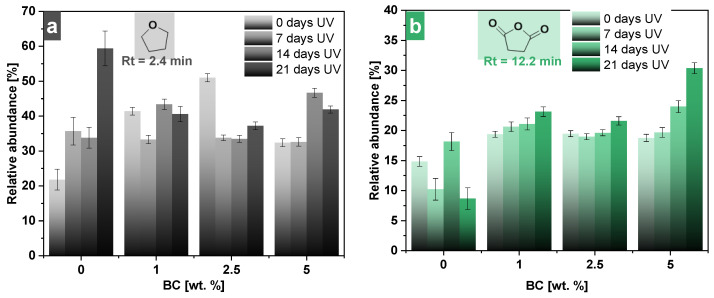

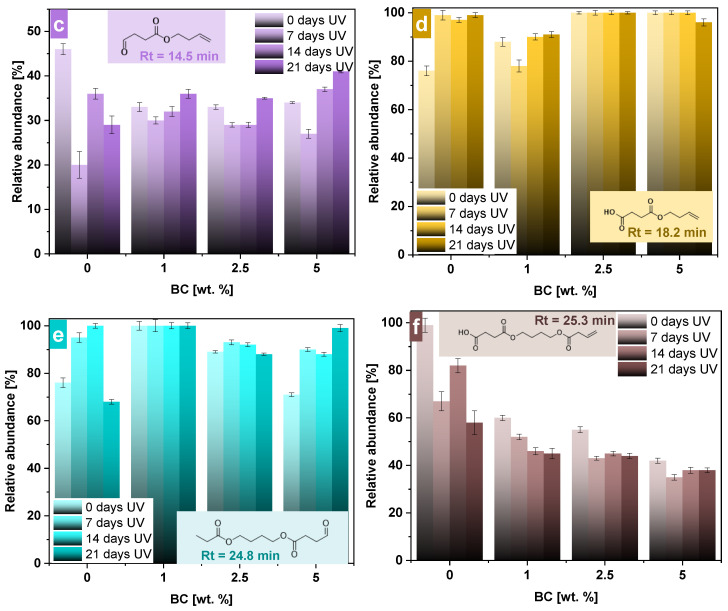
Profile of main decomposition products for different contents in BC in studied composites during UV exposure testing for 0, 1, 2, and 3 weeks. (**a**) Tetrahydrofuran; (**b**) succinic anhydride; (**c**) but-3-en-1-yl 4-oxobutanoate; (**d**) 4-(but-3-en-1-yloxy)-4-oxobutanoic acid; (**e**) 4-(propionyloxy)butyl 4-oxobutanoate; (**f**) 4-(4-(but-3-enoyloxy)butoxy)-4-oxobutanoic acid. Error bars represent standard deviation (SD) from three independent replicates (n = 3).

Concerning the abundance profiles of the main decomposition products for the different contents in BC in the studied composites during UV exposure, different patterns can be remarked from Figure 7. Interestingly, the relative abundance for the neat PBSu during UV aging displays remarkable discrepancies in contrast with PBSu/BC materials, and especially PBSu/BC 1 and 2.5 wt.%, in which the abundance of the characteristic pyrolysis compounds presented only small differences during the UV exposure. It could be assumed that the presence of biochar influences the degradation pathways by either stabilizing certain degradation products or promoting new ones, depending on its content. Despite its thermal stabilizing properties investigated in our previous work [31], biochar is suggested to possibly act as an effective UV stabilizer for PBSu, as well as for polymer matrices in general, through several mechanisms. First of all, it could absorb UV radiation, preventing it from penetrating the polymer matrix and reducing the energy available for bond breakage and photodegradation. Secondly, biochar could scavenge free radicals generated by UV exposure, neutralizing these reactive species and inhibiting further degradation processes. The physical presence of biochar particles creates a barrier effect that limits the diffusion of oxygen and other reactive species, thereby mitigating oxidative degradation. Biochar also aids in dissipating absorbed UV energy through non-destructive pathways such as thermal relaxation [14,47,48,49,50,51]. The surface chemistry of biochar, with its aromatic structure and functional groups, interacts beneficially with the polymer matrix, enhancing its stability and improving the dispersion of biochar within the polymer [52]. These interactions create a more uniform matrix that distributes the stress from UV exposure more evenly. The observed variations in peak intensities further suggest that a higher biochar content (5 wt.% in BC) significantly affects the degradation mechanisms. These findings highlight the complex interplay between UV exposure, biochar concentration, and the resulting chemical changes in the polymer matrix, underscoring the importance of biochar in enhancing the thermal and UV stability of PBSu composites.

### 3.5. Mechanical Properties and Morphological Examination

**Figure 8:**
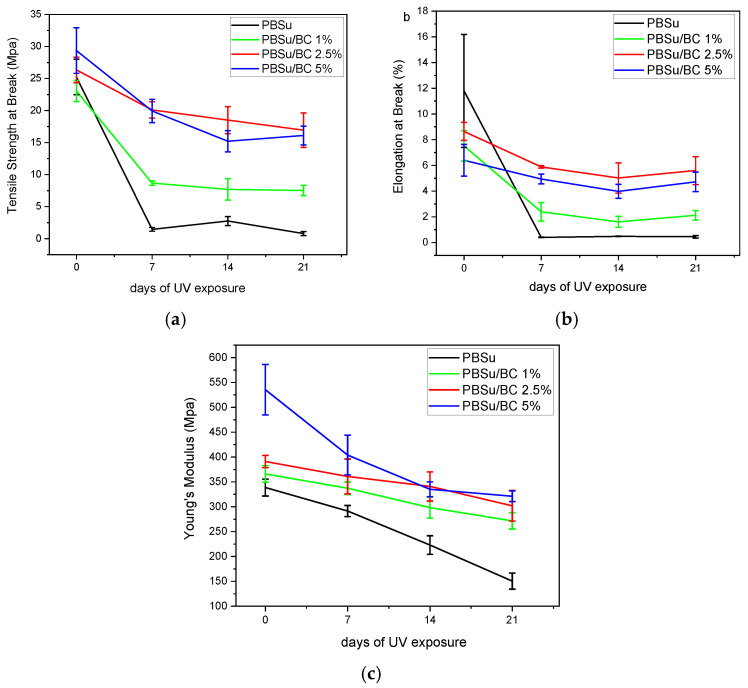
Variation in (**a**) tensile strength at break point, (**b**) elongation at break point, and (**c**) Young’s Modulus of neat PBSu and its PBSu/BC composites during different days of UV exposure. Error bars represent standard deviation (SD) from three independent replicates (n = 3).

An investigation of the mechanical properties of PBSu and its composites is essential for understanding their behavior under UV light exposure. Loss in mechanical properties of the materials is a significant indirect effect of photodegradation. Figure 8 presents the changes in mechanical properties of the materials as a function of photo-irradiation. The values of elongation at break remain at a low rate, which decrease as the exposure time increases. This happens due to the brittleness of the materials. Elongation at break (%) prior to UV exposure is the largest for neat PBSu, as expected from our previous work [21], and it depicts a remarkable drop after 21 days of UV aging. In regard to the biocomposites of PBSu/BC, they show a controlled reduction in elongation at break values. Young’s Modulus decreases continuously, indicating the severe degradation of mechanical properties after photo-irradiation. Neat PBSu presents the lowest Young’s Modulus after 21 days of exposure in UV light. Generally, all the samples presented an obvious decline in their mechanical properties, as expected from the great reduction in their molecular weight, while their crystallinity presented a fluctuating character. However, neat PBSu had a greater mechanical properties deterioration. These results are in accordance with the mechanical results of UV-irradiated PBSu samples with amine stabilizers from the study by Wenjun Liu et al. [53].

**Figure 9:**
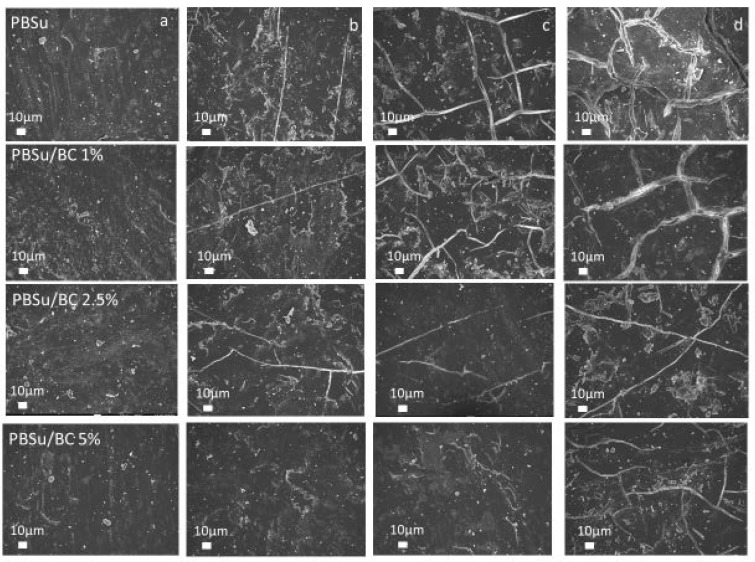
SEM micrographs of PBSu and its composites after (**a**) 0, (**b**) 7, (**c**) 14, and (**d**) 21 days of UV irradiation.

SEM analysis was conducted to reveal the morphological defects created at the surface of the studied samples during photo irradiation, since photodegradation phenomena begin from the surface of polymer films and are proceeded along the chain scissions. Figure 9 shows the surface changes in neat PBSu and composite PBSu films with 1, 2.5, and 5 wt.% biochar content before photo aging and after 7, 14, and 21 days of photo irradiation. Prior to UV exposure, all films depict smooth surface. Upon 7- and 14-day intervals of UV exposure, the surface of neat PBSu becomes rougher whereas cracks are created. In contrast, the surfaces of PBSu composites with biochar demonstrate a limited number of cracks as the biochar content increases. Neat PBSu film presented larger and deeper cracks than the relative of the composite with 5 wt.% biochar, which was less damaged. These results are in accordance with the aforementioned results, which indicated that the addition of biochar stabilizes PBSu during UV exposure.

## 4. Conclusions

The present study provides new insights into the UV aging processes of PBSu/biochar biocomposites. Investigating the UV aging effects on these biocomposites is crucial for understanding their life cycle assessment. In this context, biochar was tested as a potential UV stabilizer. Specifically, varying biochar content (1, 2.5, 5 wt.%) was incorporated into PBSu matrix via an in situ melt polycondensation procedure. The studied samples were exposed to UV irradiation for certain time intervals to assess physicochemical alternations, thermal transitions, mechanical deteriorations, and photodegradation pathways. UV irradiation deteriorated the performance of neat PBSu film, rendering it brittle after 21 days of exposure. These changes were confirmed by GPC with the significant reduction in their molecular weight, as well as by FTIR with the formation of new carbonyl and hydroxyl groups, as a result of macromolecular chain scission provoked by photo-oxidation processes. Furthermore, it is apparent that the mechanical properties exhibited an obvious deterioration, while crystallinity measurements presented a fluctuating character due to the degradation and reduction in molecular weight during UV irradiation. In contrast, incorporating biochar into the PBSu matrix protected the polymer from UV aging. As the loading of biochar increased, better UV protection was achieved. More specifically, all PBSu/BC biocomposites loaded with 1, 2.5, and 5 wt.% of biochar exhibited less mechanical deterioration after UV irradiation than the neat PBSu. Furthermore, biocomposites with 2.5 and 5 wt.% biochar presented stable crystallinity during exposure to UV irradiation, a fact that may be attributed to their absorbing UV light and, thus, inhibiting the chain scission reactions. Finally, Py–GC/MS was deemed as an essential technique for our research, enabling a detailed understanding of how biochar content influences the UV stability of PBSu biocomposites. The aforementioned results demonstrated that the use of biochar content as photostabilizer exhibited an enhancement resistance to UV irradiation. Thus, these results are crucial for the intended application of biocomposites in long-term uses, such as in agricultural mulch films.

## Figures and Tables

**Table 1:** Molecular weight of the prepared PBSu and its biochar composites during UV exposure.

Sample	M_n_ (g/mol)	M_w_ (g/mol)	PDI
PBSu 0 d	54,100	120,000	2.22
PBSu 7 d	21,600	35,900	1.66
PBSu 14 d	20,000	42,400	2.13
PBSu 21d	17,500	30,000	1.71
PBSu/BC 1% 0 d	52,700	91,200	1.73
PBSu/BC 1% 7 d	33,400	75,300	2.25
PBSu/BC 1% 14 d	32,900	73,000	2.22
PBSu/BC 1% 21 d	30,600	66,700	2.18
PBSu/BC 2.5% 0 d	48,600	126,000	2.59
PBSu/BC 2.5% 7 d	45,100	115,900	2.57
PBSu/BC 2.5% 14 d	44,000	117,000	2.66
PBSu/BC 2.5% 21 d	38,700	100,800	2.61
PBSu/BC 5% 0 d	44,000	101,500	2.3
PBSu/BC 5% 7 d	41,700	104,600	2.41
PBSu/BC 5% 14 d	37,500	87,400	2.33
PBSu/BC 5% 21 d	37,000	86,000	2.33

**Table 2:** Melting point, crystallization temperature, and degree of crystallinity of PBSu/BC composites, as calculated from DSC thermograms.

Samples	T_m_ (°C)	T_c_ (°C)	X_c_ (%)
PBSu 0 d	113	71	27
PBSu 7 d	110	72	29
PBSu 14 d	115	67	32
PBSu 21 d	102	47	21
PBSu/BC 1% 0 d	116	77	28
PBSu/BC 1% 7 d	117	77	30
PBSu/BC 1% 14 d	114	74	26
PBSu/BC 1% 21 d	114	73	26
PBSu/BC 2.5% 0 d	114	78	29
PBSu/BC 2.5% 7 d	115	78	28
PBSu/BC 2.5% 14 d	114	77	28
PBSu/BC 2.5% 21d	114	76	26
PBSu/BC 5% 0 d	115	81	28
PBSu/BC 5% 7 d	114	82	28
PBSu/BC 5% 14 d	114	82	28
PBSu/BC 5% 21 d	114	80	26

**Table 3:** Thermal decomposition products of neat PBSu during UV aging.

Retention Time (min)				Mw (g/mol)	Assigned Compound
PBSu 0d	PBSu 7d	PBSu 14d	PBSu 21d		
1.88	1.92	1.80	1.77	44	CO, CO_2_
2.43	2.51	2.52	2.40	72	2-Propenoic acid or tetrahydrofuran
6.79	7.26	6.95	7.55	86	Pent-4-en-1-ol
8.75	9.60	8.89	9.93	90	1,4-Butanediol
n.d.	n.d.	9.84	n.d.	110	Not identified
12.23	12.02	11.74	12.14	100	Succinic anhydride
12.64	12.41	12.30	12.46	118	Succinic acid
n.d.	12.82	12.66	13.01	142	But-3-en-1-yl but-3-enoate
14.43	14.49	14.44	14.56	155	But-3-en-1-yl 4-oxobutanoate
18.11	18.17	18.20	18.30	174	4-(but-3-en-1-yloxy)-4-oxobutanoic acid
n.d.	n.d.	n.d.	18.50	190	4-hydroxybutyl 4-(propionyloxy)butyl succinate
19.93	19.88	19.87	19.95	244	But-3-en-1-yl (4-hydroxybutyl) succinate
21.00	21.18	21.09	21.15	272	1,6,13-trioxacyclononadecane-7,12,14,19-tetraone
24.84	24.91	24.82	24.99	230	4-(propionyloxy)butyl 4-oxobutanoate
25.37	25.43	25.41	25.51	258	4-(4-(but-3-enoyloxy)butoxy)-4-oxobutanoic acid
n.d.	n.d.	27.39	27.48	344	1,6,11,16-tetraoxacycloicosane-2,5,12,15-tetraone or 4-(4-((4-(but-3-en-1-yloxy)-4- oxobutanoyl)oxy)butoxy)-4-oxobutanoic acid
27.91	n.d.	27.91	n.d.	399	(4-(propionyloxy)butyl) succinate bis(4-((4-oxobutanoyl)oxy)butyl)
29.45	29.49	29.45	29.54	429	bis(4-((4-oxobutanoyl)oxy)butyl) succinate

## Data Availability

All the data of this study are included in the manuscript.

## Supplementary Materials

The following supporting information can be downloaded at: https://www.mdpi.com/article/10.3390/polym16213080/s1, Table S1: Thermal decomposition products of PBSu/BC 1% during UV aging.; Table S2: Thermal decomposition products of PBSu/BC 2.5% during UV aging.; Table S3: Thermal decomposition products of PBSu/BC 5% during UV aging.
