# Supplementary material for: Biochar as a UV Stabilizer: Its Impact on the Photostability of Poly(butylene succinate) Biocomposites
Source: Polymers (Basel). 2024 Oct 31;16(21):3080. doi: 10.3390/polym16213080 (PMC11548502; doi:10.3390/polym16213080)
Supplement: Supplementary file 1 [file polymers-16-03080-s001.zip › polymers-3287663-supplementary.pdf]

Supplementary material

Biochar as a UV Stabilizer: Its Impact on the Photostability of Poly(butylene succinate) Biocomposites

Katerina Papadopoulou [1], Nina Maria Ainali [1], Ondřej Mašek [2] and Dimitrios N. Bikiaris [1,*]

[1] Laboratory of Polymer Chemistry and Technology, Department of Chemistry, Aristotle University of Thessaloniki, GR 54124 Thessaloniki, Greece; katerina_1991papa@hotmail.com (K.P.); naina@chem.auth.gr (N.M.A.)
[2] UK Biochar Research Centre, School of GeoSciences, University of Edinburgh, Alexander Crum Brown Road, Edinburgh EH9 3FF, UK; ondrej.masek@ed.ac.uk
* Correspondence: dbic@chem.auth.gr; Tel.: +30-2310997812

Supplementary materials:

Table S1. Thermal decomposition products of PBSu/BC 1% during UV aging.

Retention time (min)					
**PBSu/BC 1% 0d**	**PBSu/BC 1% 7d**	**PBSu/BC 1% 14d**	**PBSu/BC 1% 21d**	**M_w**	**Assigned compound**
1.78	1.82	1.82	1.85	44	CO, CO_2
2.39	2.19	2.44	2.41	72	2-Propenoic acid or tetrahydrofuran
6.92	7.00	7.11	7.17	86	Pent-4-en-1-ol
8.84	8.84	8.93	8.99	90	1,4-Butanediol
n.d.	n.d.	9.80	9.84	110	Not identified
12.27	12.31	12.32	12.37	100	Succinic anhydride
12.67	12.66	12.69	12.67	142	But-3-en-1-yl but-3-enoate
14.45	14.43	14.47	14.47	155	But-3-en-1-yl 4-oxobutanoate
18.07	18.09	18.29	18.32	174	4-(but-3-en-1-yloxy)-4-oxobutanoic acid
19.86	19.87	19.86	19.89	244	But-3-en-1-yl (4-hydroxybutyl) succinate
24.87	24.86	24.86	24.78	230	4-(propionyloxy)butyl 4-oxobutanoate
25.33	25.34	25.38	25.41	258	4-(4-(but-3-enoyloxy)butoxy)-4-oxobutanoic acid
27.46	27.44	27.41	27.42	399	4-((4-oxobutanoyl)oxy)butyl py y)y) (4-(propionyloxy)butyl) succinate bis(4-((4-oxobutanoyl)oxy)butyl)
29.49	29.44	29.43	29.44	429	bis(4-((4-oxobutanoyl)oxy)butyl) succinate

Table S2. Thermal decomposition products of PBSu/BC 2.5% during UV aging.

Retention time (min)					
**PBSu/BC 2.5% 0d**	**PBSu/BC 2.5% 7d**	**PBSu/BC 2.5% 14d**	**PBSu/BC 2.5% 21d**	**M_w**	**Assigned compound**
1.78	1.77	1.69	1.77	44	CO, CO_2
2.39	2.38	2.37	2.37	72	2-Propenoic acid or tetrahydrofuran
6.97	6.96	6.97	6.89	86	Pent-4-en-1-ol
8.80	8.64	8.73	8.65	90	1,4-Butanediol
12.24	12.21	12.25	12.21	100	Succinic anhydride
12.57	12.54	12.58	12.65	142	But-3-en-1-yl but-3-enoate
14.43	14.39	14.34	14.34	155	But-3-en-1-yl 4-oxobutanoate
14.52	14.54	14.53	14.53	164	1,6-dioxecane-2,5-dione Di(but-3-en-1-yl)
18.01	17.97	18.08	18.08	174	4-(but-3-en-1-yloxy)-4-oxobutanoic acid
19.80	19.83	19.80	19.84	244	But-3-en-1-yl (4-hydroxybutyl) succinate
20.98	21.01	21.04	21.00	272	1,6,13-trioxacyclononadecane-7,12,14,19-tetraone
24.80	24.73	24.71	24.75	230	4-(propionyloxy)butyl 4-oxobutanoate
25.30	25.34	25.38	25.34	258	4-(4-(but-3-enoyloxy)butoxy)-4-oxobutanoic acid
27.91	27.91	27.91	27.40	399	4-((4-oxobutanoyl)oxy)butyl py y)y) (4-(propionyloxy)butyl) succinate bis(4-((4-oxobutanoyl)oxy)butyl)
29.35	29.41	29.38	29.27	429	bis(4-((4-oxobutanoyl)oxy)butyl) succinate

Table S3. Thermal decomposition products of PBSu/BC 5% during UV aging.

Retention time (min)					
**PBSu/BC 5% 0d**	**PBSu/BC 5% 7d**	**PBSu/BC 5% 14d**	**PBSu/BC 5% 21d**	**M_w**	**Assigned compound**
1.72	1.73	1.80	1.64	44	CO, CO_2
2.32	2.37	2.35	2.33	72	2-Propenoic acid or tetrahydrofuran
7.05	7.14	7.00	6.92	86	Pent-4-en-1-ol
8.80	8.94	8.70	8.62	90	1,4-Butanediol
n.d.	9.84	9.84	9.77	110	Not identified

12.27	12.27	12.21	12.20	100	Succinic anhydride
12.60	12.61	12.60	12.51	142	But-3-en-1-yl but-3-enoate
14.32	14.41	14.41	14.36	155	But-3-en-1-yl 4-oxobutanoate
14.55	14.52	14.53	14.48	164	1,6-dioxecane-2,5-dione Di(but-3-en-1-yl)
18.06	18.05	18.11	18.03	174	4-(but-3-en-1-yloxy)-4-oxobutanoic acid
19.81	19.86	19.89	19.84	244	But-3-en-1-yl (4-hydroxybutyl) succinate
20.98	21.05	21.03	n.d.	272	1,6,13-trioxacyclononadecane-7,12,14,19-tetraone
24.74	24.77	24.73	24.69	230	4-(propionyloxy)butyl 4-oxobutanoate
25.34	25.37	25.25	25.27	258	4-(4-(but-3-enoyloxy)butoxy)-4-oxobutanoic acid
n.d.	n.d.	27.22	n.d.	344	1,6,11,16-tetraoxacycloicosane-2,5,12,15-tetraone *or* 4-(4-((4-(but-3-en-1-yloxy)-4-oxobutanoyl)oxy)butoxy)-4-oxobutanoic acid
n.d.	n.d.	n.d.	28.20	399	4-((4-oxobutanoyl)oxy)butyl py y)y) (4-(propionyloxy)butyl) succinate bis(4-((4-oxobutanoyl)oxy)butyl)
29.34	29.41	29.35	29.16	429	bis(4-((4-oxobutanoyl)oxy)butyl) succinate
